# Optimization of adeno-associated viral vector-mediated transduction of the corticospinal tract: comparison of four promoters

**DOI:** 10.1038/s41434-020-0169-1

**Published:** 2020-06-23

**Authors:** Bart Nieuwenhuis, Barbara Haenzi, Sam Hilton, Alejandro Carnicer-Lombarte, Barbara Hobo, Joost Verhaagen, James W. Fawcett

**Affiliations:** 1 John van Geest Centre for Brain Repair, Department of Clinical Neurosciences, University of Cambridge, Forvie Site, Robinson Way, Cambridge, CB2 0PY UK; 2 Laboratory for Regeneration of Sensorimotor Systems, Netherlands Institute for Neuroscience, Royal Netherlands Academy of Arts and Sciences (KNAW), Meibergdreef 47, 1105 BA Amsterdam, The Netherlands; 3 Centre for Neurogenomics and Cognitive Research, Amsterdam Neuroscience, Vrije Universiteit Amsterdam, De Boelelaan 1085, 1081 HV Amsterdam, The Netherlands; 4 Centre of Reconstructive Neuroscience, Institute of Experimental Medicine, Vídeňská 1083, 142 20 Prague 4, Czech Republic

**Keywords:** Regeneration and repair in the nervous system, Somatosensory system

## Abstract

Adeno-associated viral vectors are widely used as vehicles for gene transfer to the nervous system. The promoter and viral vector serotype are two key factors that determine the expression dynamics of the transgene. A previous comparative study has demonstrated that AAV1 displays efficient transduction of layer V corticospinal neurons, but the optimal promoter for transgene expression in corticospinal neurons has not been determined yet. In this paper, we report a side-by-side comparison between four commonly used promoters: the short CMV early enhancer/chicken β actin (sCAG), human cytomegalovirus (hCMV), mouse phosphoglycerate kinase (mPGK) and human synapsin (hSYN) promoter. Reporter constructs with each of these promoters were packaged in AAV1, and were injected in the sensorimotor cortex of rats and mice in order to transduce the corticospinal tract. Transgene expression levels and the cellular transduction profile were examined after 6 weeks. The AAV1 vectors harbouring the hCMV and sCAG promoters resulted in transgene expression in neurons, astrocytes and oligodendrocytes. The mPGK and hSYN promoters directed the strongest transgene expression. The mPGK promoter did drive expression in cortical neurons and oligodendrocytes, while transduction with AAV harbouring the hSYN promoter resulted in neuron-specific expression, including perineuronal net expressing interneurons and layer V corticospinal neurons. This promoter comparison study contributes to improve transgene delivery into the brain and spinal cord. The optimized transduction of the corticospinal tract will be beneficial for spinal cord injury research.

## Introduction

The corticospinal tract (CST) is an important descending motor pathway controlling the movement of the limbs and trunk. Damage to the CST results in paralysis. Repair of the CST is limited because this motor pathway has a low intrinsic neuronal capacity for axon regeneration. The axon regeneration capacity of corticospinal neurons can be enhanced by delivering regeneration-associated genes using gene therapy (reviewed in [1–4]). Manipulation of the CST of rats and mice is widely used to examine the neuron-intrinsic mechanisms that underlie the failure of axon regeneration.

The descending CST originates in the motor cortex. The rodent motor cortex is relatively large and covers a big area of the frontal cortex [5–7]. Layer V of the motor cortex contains pyramidal neurons, which are the main motor neurons of the CST. The axons of layer V corticospinal neurons descend via the internal capsule towards the pyramid in the rostral medulla. At the pyramidal decussation, a large proportion of the CST fibres cross over to the contralateral part of the spinal cord. The fibres that are crossing over project through the ventral part of the dorsal column to innervate all lamina of the spinal cord, mostly interneurons within lamina III–VII [8–10]. The small proportion of rodent fibres that do not cross continue ipsilaterally in the ventromedial- and lateral columns of the spinal cord to innervate interneurons of lamina III–VI [11], and motoneurons in the ventral horn [12, 13]. The CST-innervated motor neurons can stimulate muscles in the periphery to control movements of the animal.

Recombinant adeno-associated viral vectors (AAVs) are the preferred viral vectors to target neurons of the central nervous system (CNS), and have been used in clinical trials for several neurological disorders (reviewed in [14–16]), highlighting that AAV is gaining increasing acceptance as a vector for clinical gene delivery. There are different serotypes of AAVs each exhibiting different cellular tropism. The transduction efficiency of a specific cell type by an AAV vector is dependent on interaction of the viral capsid with cell surface receptors, cellular uptake and subsequent release of the vector from endosomes, trafficking to the nucleus and finally transcription and translation of the transgene (reviewed in [17–19]). As discussed above, gene therapy for axon regeneration within the CST should be aimed at layer V pyramidal neurons of the motor cortex. A direct side-by-side comparison of AAV vector serotypes (with a CMV promoter) in the CST of rats revealed that AAV1, followed by AAV5, is the most efficient serotype to transduce layer V cortical neurons in rats after direct injection [20]. Other studies that examined AAV serotypes after direct injection into the rodent cortex also found that AAV1 efficiently transduces cortical neurons [21–23].

The promoter is a major DNA regulatory element in the viral vector genome that determines the level of transgene expression and in which cells the transgene will be expressed. The choice of promoter is therefore an essential aspect of the design of AAV vectors. Furthermore, the size of the promoter is also relevant as AAVs have a maximum packaging capacity of ~4700 nucleotides. To date, a promoter that results in strong and selective expression of transgenes in layer V corticospinal neurons has not been determined by a direct side-by-side comparison.

The aim of this study was to identify a promoter that initiates strong transgene expression and is specific for cortical neurons, preferably layer V corticospinal neurons. We compared in rats and mice transgene expression under four different promoters delivered by AAV1, including the short variant of CMV early enhancer/chicken β actin (sCAG) promoter, the human CMV (hCMV) promoter, the mouse PGK (mPGK) promoter, and the human synapsin (hSYN) promoter. These four promoters were chosen because: (1) these are relatively compact promoters thus allowing for the accommodation of relatively large transgenes; (2) these promoters have been used to transduce the nervous system but their activity in the brain has not been studied in a side-by-side comparison; and (3) the specific requirement for a strong promoter in the large CST. This AAV1 promoter comparison study contributes to the optimization of AAV-mediated gene transfer to the CST and cortex.

## Materials and methods

### AAV vector plasmids

**Fig. 1:**
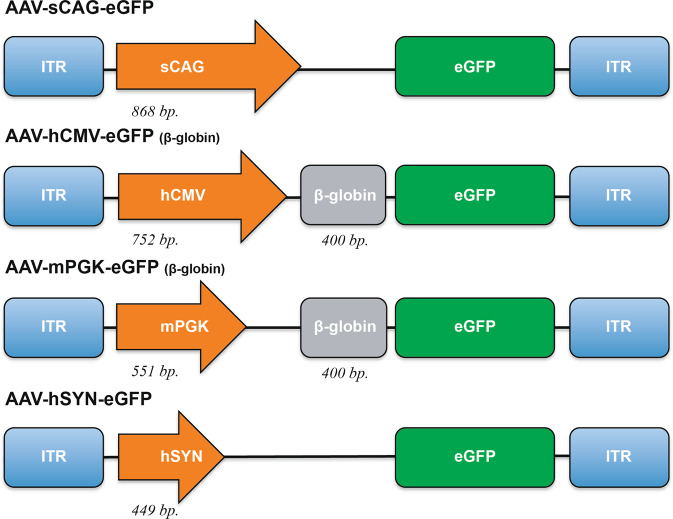
Schematic representation of adeno-associated viral vectors. Four vectors that express enhanced green fluorescent protein (eGFP, 720 bp) under different promoters. The short CAG (sCAG) promoter is 868 bp long. The human CMV (hCMV) promoter has a size of 752 bp and is upstream of a 400 bp β-globin intron. The mouse PGK (mPGK) promoter is 551 bp long and is upstream of a 400 bp β-globin intron. The shortest promoter is human SYN (hSYN) with a size of 499 bp. The packaging cassettes are flanked by inverted terminal repeats (ITR). The poly-A signals are not shown. bp base pairs.

Vector plasmids were designed to investigate the expression potential of different promoters (Fig. 1). AAV-sCAG-eGFP was provided by the laboratory of Joost Verhaagen and is described in [24]. AAV-hCMV-eGFP was a gift from Connie Cepko (Addgene plasmid # 67634). AAV-mPGK-eGFP was made by amplifying the eGFP sequence of AAV-hCMV-eGFP by using the primers 5′ GGAATTCATGGTGAGCAAGGGCGAG 3′ and 5′ AGCGCTTTACTTGTACAGCTCGTCCATG 3′ and was then cloned into an AAV-mPGK plasmid, which was a gift from Patrick Aebischer (Addgene plasmid # 24593), via HindIII-HF (NEB, R3104) and EcoRI-HF (NEB, R3101) digestion. AAV-hSYN-eGFP was made by removing the WPRE from plasmid pTRUF20B-SEW, a gift from Deniz Kirik (Lund University) described in [25], by HincII (NEB, R0103) digestion.

### Cortical neuron cultures and magnetofection

Glass bottom cell culture dishes (Greiner bio-one, 627860) were coated with 7.5 mg poly-D-lysine in 0.1 M borate buffer (pH 8.5) overnight at room temperature, and were washed with PBS the following day. Primary cortical neuron cultures were prepared from embryonic day 18 Sprague Dawley rats. The embryonic cortex was dissected in Hanks’ balanced salt solutions (HBBS) (Thermo Fischer Scientific, 14170) supplemented with a 10 mM HEPES (Thermo Fischer Scientific, 15630106) using forceps. The neurons were dissociated in 40 units of papain (Worthington, LK003176) per dissected brain that was dissolved in HBBS-HEPES for 10 min at 37 °C. Next, they were briefly rinsed with DNase (Sigma-Aldrich, D5025) and washed with HBSS-HEPES solution. The cortical tissue was carefully dissociated using different sizes of glass Pasteur pipets and the tissue was applied through a 40 μm falcon cell strainer (Thermo Fischer Scientific, 352340) to remove non-dissociated cortical tissue. The dissociated neurons were counted and ~300,000 cells were then cultured in neural Q basal medium (Globalstem, gsm9420), supplemented with 2% GS21 (Amsbio, gsm3100) and 1% glutamax (Thermo Fischer Scientific, 35050061) on the poly-D-lysine coated cell culture dishes. The cultured neurons were kept at 37 °C in a 7% CO_2_ incubator.

The cortical neurons were transfected at 10 days in vitro (DIV) using oscillating nanomagnetic transfection (magnefect nano system; nanoTherics). The vector plasmids (7 μg per dish) were mixed with 8 μl of magnetic nanoparticles (NeuroMag, NM50200) in 100 μl of culture medium without supplements and were incubated for 30 min. Afterwards the transfection mixture was topped up with 900 μl of medium, and then cell culture media in the dishes was substituted for the transfection mixture and the dishes were placed over a magnetic array, moving laterally at 2 Hz and at 0.2 mm amplitude of displacement for 30 min at 37 °C in a 7% CO_2_ incubator. After transfection, the transfection mixture was removed and the original culture medium was returned to each well. The neurons were then cultured until 14 DIV. At this time point, the neurons were fixed with 4% paraformaldehyde (PFA) in PBS for 10 min at room temperature and afterwards washed with PBS. The cells were mounted using FluorSave^TM^ reagent (Calbiochem, 345789) and by applying a 19 mm diameter round coverslip.

### Image acquisition and morphology analysis of cultured cortical neurons

Images for fluorescent intensity analyses were captured on a fluorescence microscope (DM6000 B, Leica) with a ×40-oil objective. The images were taken with the soma in the centre to be able to quantify the number of dendritic branches in series of circles with increasing radii. Semi-automated and standardized analysis of the images was performed using the MATLAB platform version 2017 and a programme called SynD [26]. SynD detects the soma by thresholding and component analysis. Afterwards, the programme detects neurites by applying a steerable filter to the image. Neurites that were not completely traced by the programme were manually extended, while spines or debris that were incorrectly identified as neurites were manually erased. The following output of SynD was used: (1) the average fluorescence of each neurite within a 100 μm distance from the soma, (2) soma fluorescence intensity, (3) soma area and (4) dendritic sholl analysis.

### Adeno-associated viral vector preparation

The small-scale production of recombinant AAVs was done as described previously [27]. The titres of AAVs were determined by quantitative PCR on the viral genomic DNA. The AAVs were titre matched to 1.42 × 10^12^ gc/ml before stereotactic injections into the sensory-motor cortex of animals.

### Animals and stereotactic injection of adeno-associated viruses in the sensory-motor cortex

A total of 20 adult female rats (Lister Hooded, Charles River Laboratories) and 20 adult female mice (C5BL/6, Charles River Laboratories) were used in this study. The animals were housed in groups and had access to water and food ad libitum in standard laboratory conditions.

**Fig. 2:**
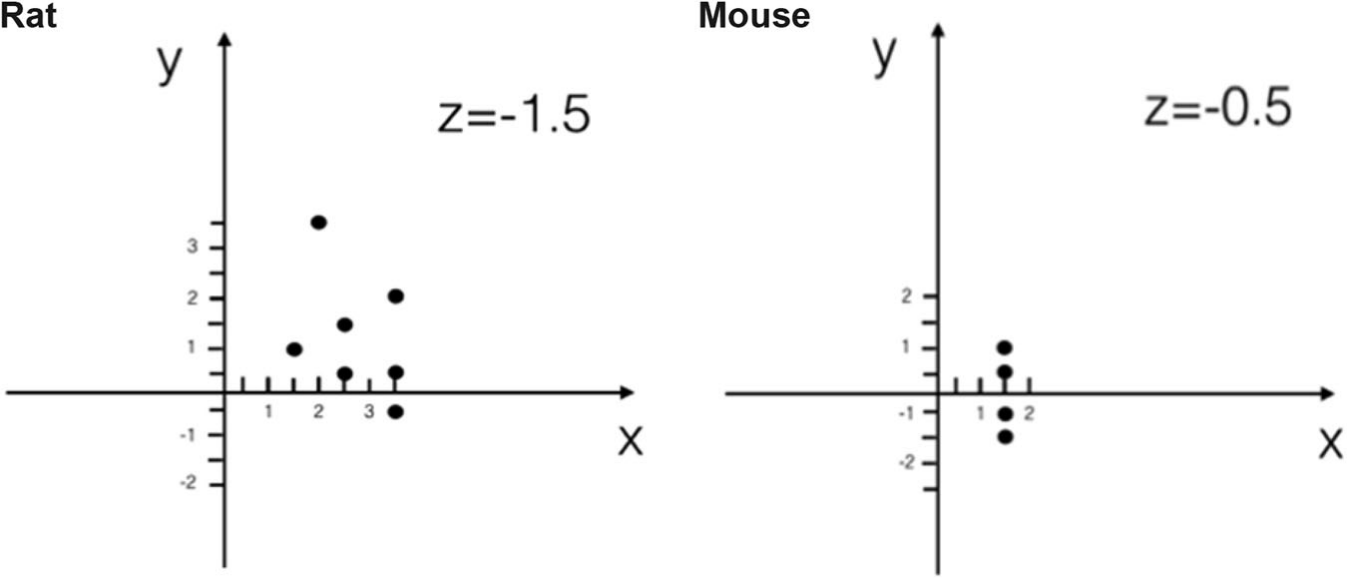
Injection coordinates for the sensory-motor cortex in the right hemisphere of rats and mice. The 0 point of the *XY* graphs represents Bregma and the coordinates were defined by their mediolateral (*x*) and anteroposterior (*y*) position relative to Bregma. Each site was injected with 0.5 μl of AAV vector. (Left) Rats were injected at seven coordinates: +1.5, +1 mm; +2, +3.5 mm; +2.5, +1.5 mm; +2.5, +0.5 mm; +3.5, +2 mm; +3.5, +0.5 mm, +3, −0.5 mm. The virus was injected at a depth of 1.5 mm (*z* = −1.5 mm). (Right) Mice were injected at four coordinates: +2, +1 mm; +2, +0.5 mm; +2, −0.5 mm; +2, −1 mm. The virus was injected at a depth of 0.5 mm (*z* = −0.5 mm).

This research has been regulated under the Animals (Scientific Procedures) Act 1986 Amendment Regulations 2012 following ethical review by the University of Cambridge Animal Welfare and Ethical Review Body. All procedures were performed using aseptic techniques. Viral vectors were stereotactically injected into the right sensory-motor cortex of mice and rats as described below. The animals were anesthetized with isoflurane (4% isoflurane in O_2_ for induction, 2% isoflurane for maintenance) delivered via a facemask and received postoperative analgesia. Body temperature was kept at roughly 36 °C via a heating pad with an anal probe. The head of the animal was shaved and the skin was disinfected. The animal was next placed in the stereotaxic frame (David Kopf Instruments) were a midline incision was made to expose the skull and holes were drilled at coordinates that cover the right sensory-motor cortex (Fig. 2). A Hamilton syringe with needle was positioned through the generated holes and was lowered into the brain by 0.5 mm for mice and 1.5 mm for rats. Thereafter, 0.5 μl of AAV vector was injected in each hole at a rate of 0.2 μl per minute (total virus volume of 2 μl per mice and 3.5 μl per rat) using a Micro4 micro syringe pump controller. The needle was slowly removed 2 min after injection to allow proper diffusion of the virus and avoid unwanted leaking. Afterwards, the skin was sutured and the animals were recovered at 37 °C before they were returned to standard housing.

One rat (out of the 40 injected animals in total) that was injected with AAV1-mPGK-eGFP was excluded from the study because the stereotactic injections poorly covered the sensory-motor cortex.

### Histology

Six weeks after the injection of AAV vectors, animals were euthanized by injecting an overdose of Euthatal followed by transcardial perfusion with ice cold PBS followed by ice cold 4% PFA in PBS. The brain and spinal cords were collected and stored in 4% PFA in PBS for 24 h at 4 °C. Afterwards, the tissue was stored in 30% sucrose in PBS with 0.1% sodium azide at 4 °C until further use. The cerebrum was cut into 40 μm tick coronal sections and the spinal cords were cut in transverse sections with a thickness of 40 μm using a freezing microtome. The cerebrum tissue was collected in 12 series creating an anatomical distance of 480 μm between each section in one well (in other words, section 1 was placed into well 1, section 2 was placed into well 2, and this was done until section 12. Afterwards, this process was repeated). The tissue was kept in a 12-well plate containing PBS and 0.1% sodium azide at 4 °C in the dark until further use.

### Immunohistochemistry

The sectioned brains and spinal cords were washed with PBS. Then the tissue was permeabilized and blocked by applying 0.3% Triton X-100 (Sigma, X-100) and 10% goat serum (Sigma-Aldrich, G9023) in PBS for 2 h at room temperature. The tissue was then incubated with primary antibodies that were diluted in above-mentioned blocking detergent at 4 °C overnight. The primary antibodies used were anti-eGFP (A10262, 1:1000, Thermo Fischer Scientific or Ab290, 1:1000, Abcam), anti-NeuN (ABN91, 1:500, Merck), anti-Aggrecan (AB1013, 1:400, Merck), anti-Glial Fibrillary Acidic protein (Z0334, 1:500, Dako), anti-Iba1 (019–19741, 1:1000, Wako), anti-APC clone CC1 (OP80, 1:300, Merck), anti-Olig-2 (AB9610, 1:500, Merck). The perineuronal net (PNN) marker Wisteria floribunda agglutinin (WFA) was labelled by applying biotin-conjugated lectin (L1516, 4 μg/ml, Merck) instead of a primary antibody. Afterwards, the tissues were washed and incubated in Alexa Fluor-conjugated secondary antibodies that were diluted in 0.3% Triton X-100 and 10% goat serum in PBS for 2 h at room temperature in the dark. The secondary antibodies used were anti-chicken IgY conjugated to Alexa Fluor 488 (A-11039, 1:500, Thermo Fischer Scientific), anti-chicken IgY conjugated to Alexa Fluor 568 (A-11041, 1:500, Thermo Fischer Scientific), anti-rabbit IgG conjugated Alexa Fluor 488 (A-11008, 1:500, Thermo Fischer Scientific), anti-rabbit IgG conjugated Alexa Fluor 568 (A-11011, 1:500, Thermo Fischer Scientific), anti-rabbit IgG conjugated Alexa Fluor 647 (A-21245, 1:500, Thermo Fischer Scientific), anti-mouse IgG2b conjugated Alexa Fluor 568 (A21144, 1:500, Thermo Fischer Scientific). WFA was visualized by applying streptavidin conjugated Alexa Fluor 568 (S11226, 1:500, Thermo Fischer Scientific) to the tissue. After washing, the free-floating sections were mounted using FluorSave^TM^ reagent (Calbiochem, 345789) on glass imaging slides.

### Microscopy

Cultured cortical neurons were imaged on a fluorescence microscope (DM6000 B, Leica) with a ×40-oil objective using Leica software for quantifications. Representative images of cultured cortical neurons were taken using an SP5 confocal microscope with a ×40-oil objective and a 2.5 digital zoom factor using Leica software. Fluorescent tissue slides stained for NeuN were scanned on a Zeiss AxioScan Z1 (Histopathology core facility in Cancer Research UK—Cambridge institute) using a ×20 light objective. Images containing the regions of interest were exported in TIFF format. All images within an experiment were taken with identical settings when the eGFP intensity was quantified. Representative images for astrocytes, microglia, the oligodendrocytes lineage, neurons with PNNs and the dCST were taken using laser-scanning confocal microscopy (Leica, TCS SPE, DMI4000B).

### Quantification of histological samples

The mean area of transduction per section was measured in ImageJ by manually outlining the area that contained eGFP-positive staining. The transduction area reported for each animal is the average taken from two sections per animal with the biggest transduction area.

The number of transduced cortical neurons (eGFP+ NeuN+ cells) and the mean eGFP intensity per transduced neuron was measured using a custom-made ImageJ Macro (Supplementary Table 1) and data-processing spreadsheet to process the ImageJ output (Supplementary Data 1). Importantly, the numbers reported for each animal are the average taken from two sections with the biggest transduction area. The ImageJ macro automatically detects round cells that are present in the red channel of an image (e.g. NeuN+ cells) and then measures the fluorescent intensity of all marked cells in the green channel (e.g. eGFP). Afterwards, manual eGFP intensity measurements of three non-transduced cells were done to set a threshold for the background signal in non-transduced neurons. In addition, manual eGFP intensity measurements were taken in between multiple transduced somata within the region of interest to set the background threshold within the transduced area. After applying the Macro and completion of the two background measurements, the number of transduced neurons and the mean eGFP intensity per transduced neuron were calculated in the data-processing spreadsheet. Cells were automatically classified as transduced cortical neurons when the eGFP fluorescent intensity was higher than an arbitrary limit of 30% above the average background intensity of the transduced area. Because each brain was cut into a series of 12, the number of transduced cortical neurons of each image was multiplied by 12 to obtain the total expected number of transduced neurons per section covering ~480 μm in anteroposterior direction. The mean eGFP intensity of transduced cortical neurons was calculated by taking the average eGFP intensity of these neurons and afterwards subtracting the average eGFP intensity of three non-transduced cells chosen at random within the same section. To examine whether our analysis pipeline was correctly calibrated, we performed manual analysis of ten random cropped images of AAV transduced cortical tissue and compared this with the new analysis method. There was no statistically significant difference in the number of transduced cortical neurons between the manual counts (defined as 100%) and the ImageJ analysis pipeline (112 ± 9.5% fraction of normalized cells) (Supplementary Fig. 1), validating this quantification method for the number of transduced cortical neurons.

The number of eGFP-containing axons in the dCST was manually counted by using microscope eyepiece reticles (Pyser Optics, NE11A—01B26210) and a standard fluorescence microscope (DM6000 B, Leica) with ×63-oil objective. The squared grid covered a region of 80 × 80 μm with this magnification. Axon transduction for each animal was determined by taking the average number of green fluorescent axons in three counting grids. This average number was multiplied by factor 10 or 4 for rats or mice, respectively, to cover the transduced dCST at the cervical spinal cord. Confocal images with ×63-oil objective were taken to measure the eGFP intensity of the transduced axons. The mean eGFP intensity was determined by taking the average fluorescent intensity of ten axons and subtracting one background measurement.

The number of transduced PNN-bearing interneurons was manually counted from images taken with a standard fluorescence microscope (DM6000 B, Leica) with ×40-oil objective. The images covered a physical length of 320 × 240 μm. The number of transduced PNN-bearing interneurons for each animal was determined by taking the average number of eGFP+ cells that were surrounded by WFA (Alexa 568) and/or Aggrecan (Alexa 647) staining in six images of two sections with the biggest area of AAV transduction.

The number of transduced astrocytes and microglia were manually counted by using microscope eyepiece reticles (Pyser Optics, NE10A—01B26208) and a standard fluorescence microscope (DM6000 B, Leica) with ×40-oil objective. The squared grid covered a region of 250 × 250 μm with this magnification. The transduced cells were visualised by using a filter that permits both green (Alexa 488) and red (Alexa 568) fluorescent light through the eyepiece. The number of transduced cells per ROI for each animal was calculated by taking the average number of transduced cells in three counting grids of two sections with the biggest area of AAV transduction.

The number of transduced cells from the oligodendrocyte lineage was manually counted from images taken with a standard fluorescence microscope (DM6000 B, Leica) with ×63-oil objective. The images covered a physical length of 203 × 152 μm. The number of transduced cells for each animal was determined by taking the average of the number of Olig2+ eGFP+ cells in three images of the section with the largest area of AAV transduction in the corpus callosum.

### Statistical analysis

Statistical analysis was performed by using Graphpad Prism 7 for Mac OS X. All statistical tests and parameters are mentioned in the figure legends. The graphs represent the average for each condition together with the individual data points for each animal, rather than error bars, because of the relatively small *N* numbers per group used in this study [28]. The Shapiro–Wilk test was used to test normality and the Brown–Forsythe test was performed to examine equality of group variances. Comparisons between two experimental groups (e.g. AAV1 and AAV5) were done using an unpaired *t*-test or Mann–Whitey test depending on the distribution of the variables. Comparisons between more than two experimental groups (e.g. four promoters) and one measured variable were done using One-way ANOVA and Tukey’s multiple comparison test or Kruskal–Wallis test and Dunn’s multiple comparison test depending on the distribution of variances. For the sholl analysis of neurite branches, we used a repeated measure ANOVA test since an interaction between the two independent variables ‘number of branches’ and ‘distance from soma’ is examined between four experimental groups.

## Results

### Validation of gene expression from the AAV vector plasmids in cortical neurons in vitro

**Fig. 3:**
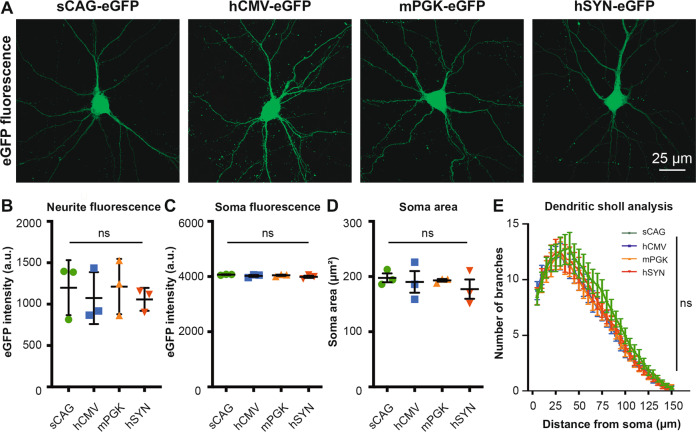
Comparison of eGFP expression driven by each of the four promoters in cultured cortical neurons transfected with AAV vector plasmids. Intrinsic eGFP was measured. **a** Representative images of 14 DIV cortical neurons that were transfected with four different plasmids: sCAG-eGFP, hCMV-eGFP, mPGK-eGFP, hSYN-eGFP. **b** Quantifications of the mean eGFP intensity in neurites (df = 3(8), *F* = 0.23, *p* > 0.05, ANOVA). **c** Quantification of the mean eGFP intensity in the soma (df = 3(8), *F* = 1.39, *p* > 0.05, ANOVA). **d** Quantification of the soma area (df = 3(8), *F* = 0.41, *p* > 0.05, ANOVA). **e** Sholl analysis of neurite branches (df = 3(8), *F* = 0.52, *p* > 0.05, Repeated measure ANOVA, *n* = 3 per group). Data are shown as average ± SEM. The results are obtained from three independent experiments (*n* = 3) and a total of 45 transfected neurons were analysed for each condition. Images were taken with identical microscope settings between the experimental groups. Ns not statistically significant.

The plasmids were transfected in cortical neurons in vitro, before AAV vectors were produced, to validate that the cloned plasmids are functional in these cells. Each of the four plasmids resulted in eGFP expression after transfection (Fig. 3a), validating the functionality of the AAV vector plasmids in primary cortical neurons. The strength of the four promoters in vitro was also determined by measuring the intensity of eGFP. Four days after the transfection, the eGFP intensity was similar between the sCAG, hCMV, mPGK and hSYN promoters (Fig. 3b, c). The eGFP intensity is high in the soma (Fig. 3c) as the fluorescent protein is synthesized here after plasmid transfection. As expected, the investigated primary cortical neurons had a similar neuronal morphology in all experimental groups (Fig. 3d, e).

### Efficiency of the four promoters to express eGFP in cortical neurons in vivo

A previous study comparing multiple AAV serotypes harbouring a CMV promoter showed that AAV1 is the most efficient serotype to transduce the CST in rats [20]. We compared AAVs serotypes 1 and 5 with an hSYN promoter and confirmed that AAV1 is superior over AAV5 to transduce cortical neurons (Supplementary Fig. 2) and layer V corticospinal neurons (Supplementary Fig. 3) in rats and mice. We next decided to determine the optimal promoter for transgene expression in the sensorimotor cortex and CST using the AAV1 serotype. AAV1-sCAG-eGFP, AAV1-hCMV-eGFP, AAV1-mPGK-eGFP and AAV1-hSYN-eGFP were injected into the right sensory-motor cortex of rats and mice for this purpose (see Fig. 2 for injection sites). The transduction area, the number of transduced neurons and the expression intensity of eGFP were measured 6 weeks after AAV injection.

**Fig. 4:**
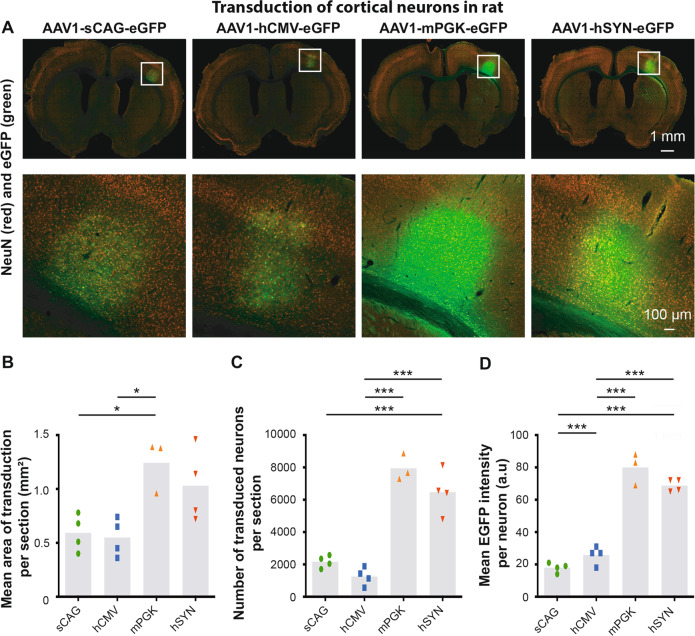
Neuronal transduction efficiency and mean eGFP intensity of four promoters in the sensory-motor cortex of rats. **a** NeuN and eGFP staining in the cortex of AAV1-sCAG-eGFP, AAV1-hCMV-eGFP, AAV1-mPGK-eGFP and AAV1-hSYN-eGFP injected rats. **b** Quantification of the mean area of transduction in 40 μm thick sections (df = 3(11), *F* = 6.88, *p* < 0.01, ANOVA with Tukey’s multiple comparison test). **c** Quantification of the number of transduced NeuN+ cells. eGFP+ NeuN+ cells were detected using the analysis pipeline and neurons were considered as transduced when the eGFP intensity was 1.3× higher than the eGFP to background intensity ratio (df = 3(11), *F* = 50.4, *p* < 0.001, ANOVA with Tukey’s multiple comparison test). **d** Quantification of the mean eGFP intensity per transduced neuron (df = 3(12), *F* = 107, *p* < 0.001, ANOVA with Tukey’s multiple comparison test). The grey bars depict the averages and each dot represents the mean value of one animal. A total of four rats were analysed for the sCAG, hCMV and hSYN conditions, while three rats were analysed for the mPGK condition. Images were taken with identical microscope settings between the experimental groups. *p < 0.05; **p < 0.01; ***p < 0.001.

In rats (Fig. 4a), the mean area of transduction in cortical sections was significantly different between the four promoters. The AAV1 vectors with the mPGK and hSYN promoter had an average transduction area of ~1 mm^2^ (1.24 ± 0.14 and 1.03 ± 0.17 mm^2^, respectively), whereas the vectors with sCAG and hCMV transduced an area of 0.55 mm^2^ (0.59 ± 0.09 and 0.55 ± 0.09 mm^2^, respectively) (Fig. 4b). A difference between the four experimental groups was also observed when analysing the number of transduced neurons. The AAV1 vector with mPGK and hSYN promoters transduced on average the highest number of cortical neurons (7950 ± 477 and 6473 ± 682 NeuN+ cells per section, respectively) followed by sCAG and hCMV (2172 ± 189 and 1256 ± 277 NeuN+ cells per section, respectively) (Fig. 4c). Importantly, not only the number of transduced neurons was higher with the mPGK and hSYN promoters but these promoters also resulted in significantly higher levels of eGFP expression in cortical neurons (80 ± 6 and 69 ± 3 a.u., respectively) compared with the sCAG and hCMV promoters (18 ± 2 and 26 ± 3 a.u., respectively) (Fig. 4d).

**Fig. 5:**
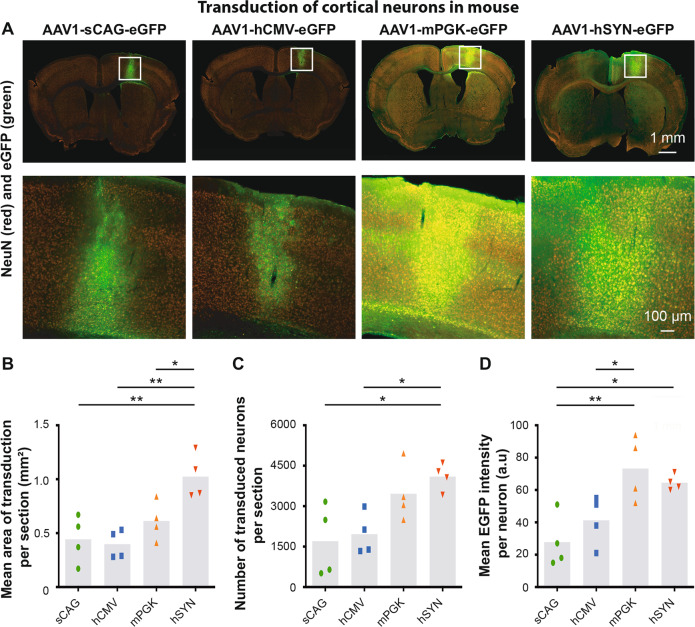
Neuronal transduction efficiency and mean eGFP intensity of four promoters in the sensory-motor cortex of mice. **a** NeuN and eGFP staining in the cortex of AAV1-sCAG-eGFP, AAV1-hCMV-eGFP, AAV1-mPGK-eGFP and AAV1-SYN-eGFP injected mice. **b** Quantification of the mean area of transduction in 40 μm thick sections (df = 3(12), *F* = 9.29, *p* < 0.01, ANOVA with Tukey’s multiple comparison test). **c** Quantification of the number of transduced NeuN+ cells. eGFP+ NeuN+ cells were detected using the analysis pipeline and neurons were considered as transduced when the eGFP intensity was 1.3× higher than the eGFP to background intensity ratio (df = 3(12), *F* = 5.74, *p* < 0.05, ANOVA with Tukey’s multiple comparison test). **d** Quantification of the mean eGFP intensity per transduced neuron (df = 3(12), *F* = 7.58, *p* < 0.01, ANOVA with Tukey’s multiple comparison test). The grey bars depict the averages and each dot represents the mean value of one animal. A total of four mice were analysed for each condition. Images were taken with identical microscope settings between the experimental groups. *p < 0.05; **p < 0.01.

In mice (Fig. 5a), the mean area of transduction in cortical sections was also statistically different between the four promoters. The AAV1 vector carrying the hSYN promoter (1.03 ± 0.10 mm^2^) had the biggest area of transduction compared with the other promoters (mPGK, 0.62 ± 0.09 mm^2^; sCAG, 0.44 ± 0.11 mm^2^; hCMV, 0.40 ± 0.07 mm^2^) (Fig. 5b). The vector with the hSYN promoter transduced the highest number of cortical neurons (4098 ± 252 NeuN+ cells per section), while the vector with the mPGK promoter resulted in the second highest mean number of transduced neurons (3464 ± 530 NeuN+ cells per section). This was followed by sCAG and hCMV (1704 ± 665 and 1962 ± 387 NeuN+ cells per section, respectively) (Fig. 5c). Importantly, the eGFP intensity was also different between the four promoters. The AAV vectors with the mPGK and hSYN promoters expressed eGFP with the highest intensity in neurons (73 ± 10 and 65 ± 2 a.u., respectively). The vectors with the sCAG and hCMV promoters had relatively low eGFP intensity in cortical neurons (28 ± 8 and 42 ± 8 a.u., respectively) (Fig. 5d).

In summary, the most efficient promoters for AAV1-mediated transgene expression in cortical neurons are mPGK and hSYN in rats and mice.

### Efficiency of the four promoters to transduce layer V corticospinal neurons in vivo

**Fig. 6:**
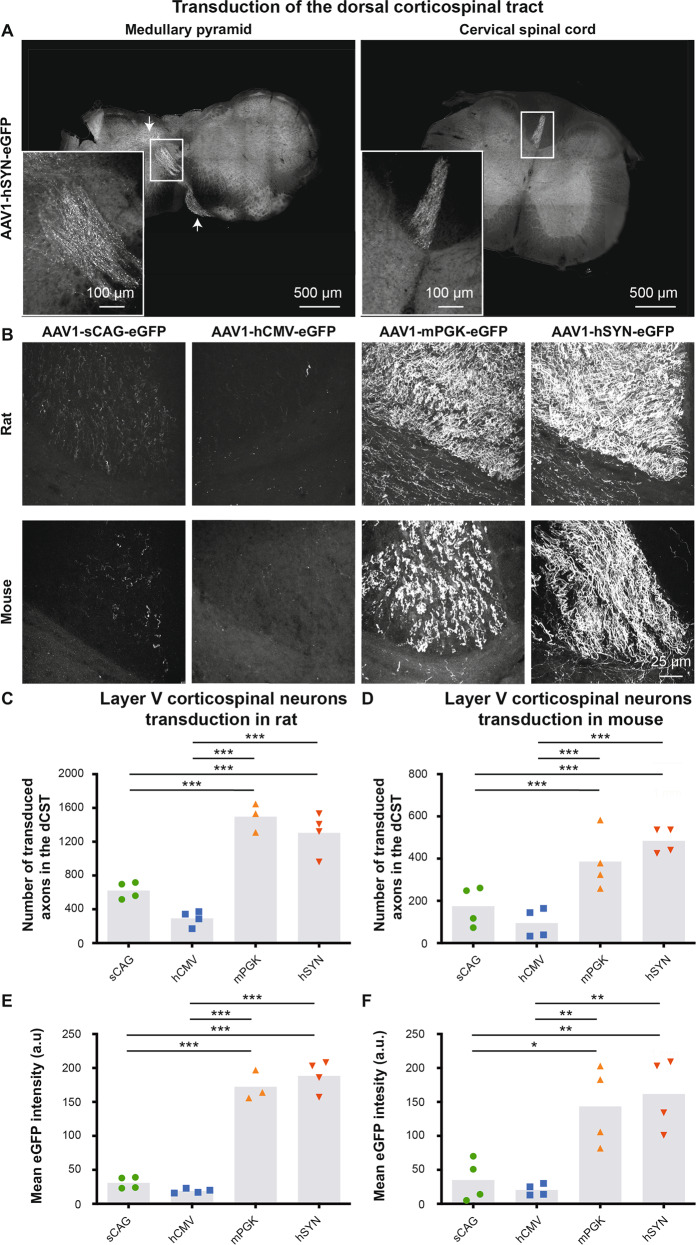
Transduction efficiency of AAV1 with four different promoters in layer V corticospinal neurons measured by quantifying their eGFP+ axons in the spinal cord at cervical level 4. The sections were stained for eGFP. **a** Transduction of the CST in a mouse injected with AAV1-hSYN-eGFP. eGFP-positive fibres are found crossing at the level of the medullary pyramid from the right to left brain hemisphere (left), and the axonal fibres are found in the left dorsal columns of the cervical spinal cord (right). **b** eGFP-positive axons in the dCST of AAV1-sCAG-eGFP, AAV1-hCMV-eGFP, AAV1-mPGK-eGFP and AAV1-hSYN-eGFP injected rats and mice. **c**, **d** Quantification of the number of transduced axons in rats (df = 3(11), *F* = 43.7, *p* < 0.001, ANOVA with Tukey’s multiple comparison test) and mice (df = 3(12), *F* = 14.2, *p* < 0.001, ANOVA with Tukey’s multiple comparison test). **e**, **f** Quantification of the mean eGFP intensity of the axon in rats (df = 3(11), *F* = 121, *p* < 0.001, ANOVA with Tukey’s multiple comparison test) and mice (df = 3(12), *F* = 11.7, *p* < 0.001, ANOVA with Tukey’s multiple comparison test). The overview images were taken with a tile-scanning epifluorescence microscope, while the zoom-in pictures were taken using a confocal microscope. The grey bars depict the averages and each dot represents the mean value of one animal. Images were taken with identical microscope settings between the eight experimental groups. *p < 0.05; **p < 0.01; ***p < 0.001.

After confirming successful transduction of neurons in the cortex, we examined whether the AAVs also transduce layer V cortical neurons that give rise to the CST. To highlight successful transduction of the CST, representative images at the medullary pyramid and cervical spinal cord were taken from tissue injected with AAV1-hSYN-eGFP. As expected, eGFP-positive axons were found to be crossing at the pyramidal decussation and descending into the left dorsal columns of the spinal cord (Fig. 6a). Transduced dCST collaterals were visible in various lamina of the spinal cord, and eGFP-positive axons were also observed in the ipsilateral ventral CST component (Supplementary Fig. 4). To obtain the transduction efficiency of layer V corticospinal neurons by AAV1 vectors with different promoters, the number of eGFP-expressing axons and fluorescent intensities were measured in the dorsal column of transverse sections of the cervical spinal cord.

In rats (Fig. 6b, c), mPGK and hSYN had the best transduction of layer V cortical neurons as 1497 ± 99 and 1305 ± 123 axons, respectively, were detected in the dorsal column of the cervical spinal cord contralateral to the injected sensory-motor cortex. The sCAG group counted 623 ± 50 green axons, and hCMV resulted on average in 293 ± 49 eGFP-positive fibres in the spinal cord. The fluorescent intensity of the transduced axons was also highest with the mPGK and hSYN promoters (172 ± 12.5 and 189 ± 11.5 a.u., respectively), and lowest with the sCAG and hCMV promoters (31 ± 4.3 and 19 ± 1.6 a.u., respectively) (Fig. 6b, e).

In mice (Fig. 6b, d), 484 ± 30 and 386 ± 70 axons were fluorescent in the spinal cord in the mPGK and hSYN groups, respectively. The sCAG and hCMV promoters had 175 ± 47 and 95 ± 34 axons containing eGFP, respectively. The fluorescent intensity of the transduced axons was higher in the mPGK and hSYN conditions (144 ± 29.3 and 162 ± 26.4 a.u., respectively) compared with sCAG and hCMV (31 ± 4.3 and 19 ± 1.6 a.u., respectively) (Fig. 6b, f).

The transduction efficiency and fluorescent intensities of the AAV1 vector carrying the mPGK and hSYN promoters were not statistically different indicating that these two promoters are equally strong in both rats and mice. Fewer and less bright eGFP-positive axons were observed in the groups that contained the AAV1 vector harbouring the sCAG and hCMV promoters indicating that these promoters are less efficient to target the CST in vivo.

### Efficiency of the four promoters to transduce cortical neurons with PNNs in vivo

**Fig. 7:**
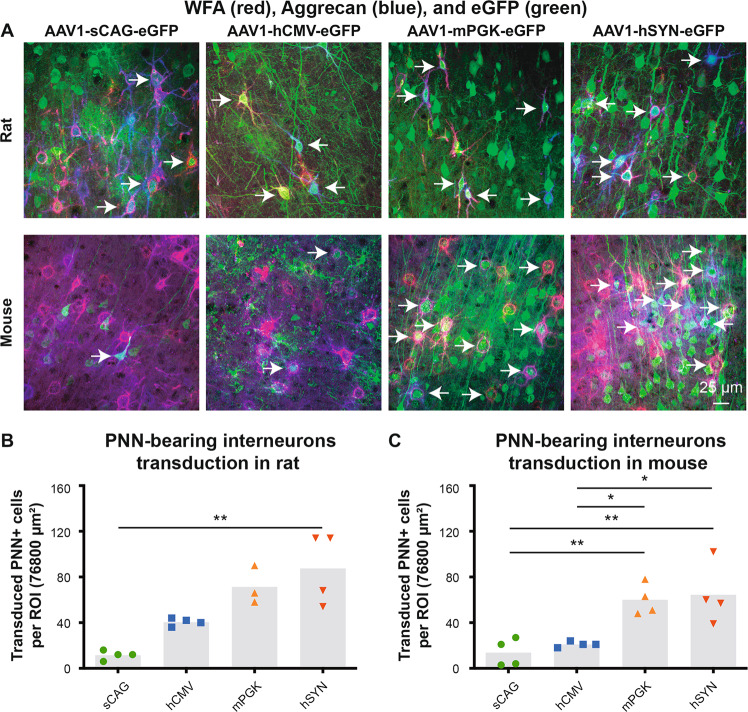
Transduction efficiency of PNN-bearing interneurons depending on the promoters sCAG, hCMV, mPGK and hSYN. **a** WFA (red), Aggrecan (blue) and eGFP (green) staining in the cortex of AAV1-sCAG-eGFP, AAV1-hCMV-eGFP, AAV1-mPGK-eGFP and AAV1-hSYN-eGFP injected rats and mice. **b**, **c** Quantification of the number of transduced PNN-bearing interneurons in rats (*p* < 0.001, Kruskal–Wallis test with Dunn’s multiple comparison test) and mice (df = 3(12), *F* = 10.4, *p* < 0.01, ANOVA with Tukey’s multiple comparison test). The grey bars depict the averages and each dot represents the mean value of one animal. Arrows highlight transduced PNN-bearing interneurons. Microscope settings for the eGFP intensity are not identical between the experimental groups in order to improve the visualisation of transduced PNN-bearing neurons; the intensity settings for WFA and aggrecan are identical throughout the representative images. *p < 0.05; **p < 0.01.

We also investigated whether PNN-bearing interneurons, another important class of cortical neurons, were transduced by the viral vectors injected in the sensorimotor cortex. The transduction of these neurons was determined by counting the number of eGFP-positive cells that were covered by PNNs, which were visualized by immunohistochemistry for WFA and aggrecan. The AAV1 serotype with all four promoters were able to transduce interneurons with PNNs in rats and mice (Fig. 7a). The promoters sCAG (12 ± 2 and 14 ± 6) and hCMV (41 ± 2 and 21 ± 1) had eGFP expression in a relatively low number of PPN-bearing interneurons (Fig. 7b, c). The mPGK (71 ± 10 and 60 ± 7) and hSYN (88 ± 16 and 65 ± 13) promoters had the highest transduction of this class of neuron (Fig. 7b, c).

### Expression of eGFP in the oligodendrocyte lineage depending on the promoter in vivo

**Fig. 8:**
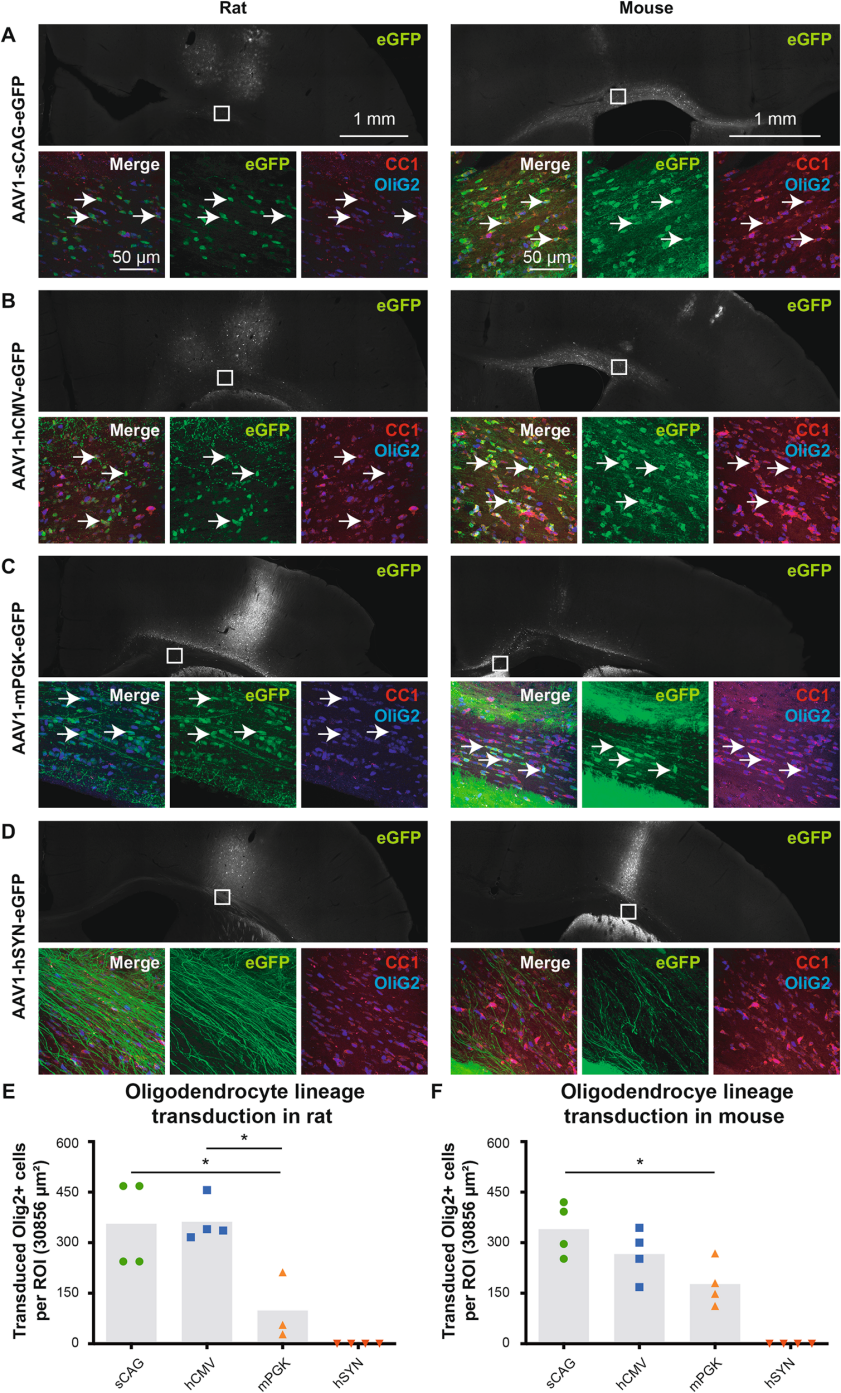
Expression of eGFP in the oligodendrocyte lineage with AAV1 and the promoters sCAG, hCMV and mPGK, but not with hSYN. The upper panels illustrate an overview of the right cerebral hemisphere that was injected with AAV1 and four different promoters expressing eGFP. The lower panels are magnification images taken at the inset in the upper panels showing a portion of the corpus callosum. The 40 μm thick brain sections were stained for clone CC1 (red), Olig2 (blue) and eGFP (green) in both rats (left) and mice (right). The experimental conditions were AAV1-sCAG-eGFP (**a**), AAV-hCMV-eGFP (**b**), AAV-mPGK-eGFP (**c**) and AAV-hSYN-eGFP (**d**). **e**, **f** Quantification of the number of transduced Olig2+ cells in rats (df = 2(8), *F* = 7.22, *p* < 0.05, ANOVA with Tukey’s multiple comparison test for sCAG vs hCMV vs mPGK) and mice (df = 2(9), *F* = 4.88, *p* < 0.05, ANOVA with Tukey’s multiple comparison test for sCAG vs hCMV vs mPGK). Arrows highlight examples of transduced cells in the oligodendrocyte lineage for sCAG, hCMV and mPGK, while no transduced oligodendrocytes are in the hSYN experimental group. Microscope settings for the eGFP intensity are not identical between the experimental groups in order to improve the visualisation of the transduced oligodendrocyte lineage; the intensity settings for CC1 and Olig2 are identical throughout the representative images. *p < 0.05.

The stereotactic injections of AAVs also resulted in transgene expression in the corpus callosum area; the transduction of the oligodendrocyte lineage was therefore examined as well. In our experiments, the clone CC1 antibody resulted in poor visualization of matured oligodendrocytes in rats (Fig. 8—left) and relatively high background staining in the corpus callosum of mice (Fig. 8—right). The anti-Olig2 antibody that labels the entire oligodendrocyte lineage was relatively consistent in both animal species (Fig. 8a–d), and was therefore used to quantify the number of transduced cells. The injection of AAV1 with sCAG (Fig. 8a), hCMV (Fig. 8b) and mPGK (Fig. 8c) promoters resulted in eGFP expression in the oligodendrocyte lineage in the corpus callosum of rats and mice. The sCAG (356 ± 65 and 340 ± 40) and hCMV (362 ± 32 and 266 ± 38) promoters transduced the highest number of OliG2-positive cells, while the mPGK promoter (99 ± 57 and 177 ± 33) had relatively few cells transduced in the corpus callosum (Fig. 8e, f). AAV1 with hSYN promoter did not transduce cells from the oligodendrocyte lineage, but clear bundles of eGFP+ axons were visible in the corpus callosum (Fig. 8d–f).

### Expression of eGFP in astrocytes depending on the promoter in vivo

**Fig. 9:**
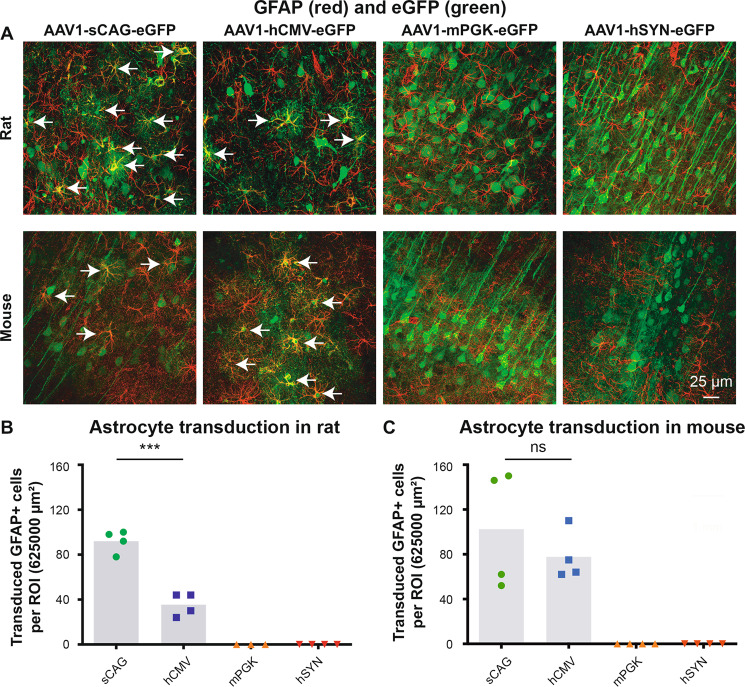
Expression of eGFP in astrocytes with sCAG and hCMV promoters, but not with mPGK and hSYN promoters. **a** GFAP (red) and eGFP (green) staining in the cortex of AAV1-sCAG-eGFP, AAV1-hCMV-eGFP, AAV1-mPGK-eGFP and AAV1-hSYN-eGFP injected rats and mice. **b**, **c** Quantification of the number of transduced GFAP+ cells in rats (df = 6, *t* = 7.97, *p* < 0.001, Student’s *t* test for sCAG vs hCMV) and mice (df = 6, *t* = 0.87, *p* > 0.05, Student’s *t* test for sCAG vs hCMV). The grey bars depict the averages and each dot represents the mean value of one animal. Arrows highlight transduced astrocytes. Microscope settings for the eGFP intensity are not identical between the experimental groups in order to improve the visualisation of transduced astrocytes; the intensity setting for GFAP is identical throughout the representative images. ns not significantly different; ***p < 0.001.

The transduction of astrocytes was determined by counting the number of GFAP-positive cells in the cortex that expressed eGFP. Astrocyte transduction was determined for the AAV1-sCAG-eGFP, AAV1-hCMV-eGFP, AAV1-mPGK-eGFP and AAV1-hSYN-eGFP transduced brains (Fig. 9a). The viral vectors containing the sCAG (92 ± 5 and 103 ± 26) and hCMV (36 ± 5 and 78 ± 11) promoter induced eGFP expression in astrocytes within the injected cerebral hemispheres of rats and mice (Fig. 9b, c). In contrast, the AAV1 vectors harbouring the mPGK and hSYN promoter did not result in eGFP expression in astrocytes for rats and mice (Fig. 9b, c).

### No transduction of microglia using AAV1 with sCAG, hCMV, mPGK and hSYN promoters in vivo

**Fig. 10:**
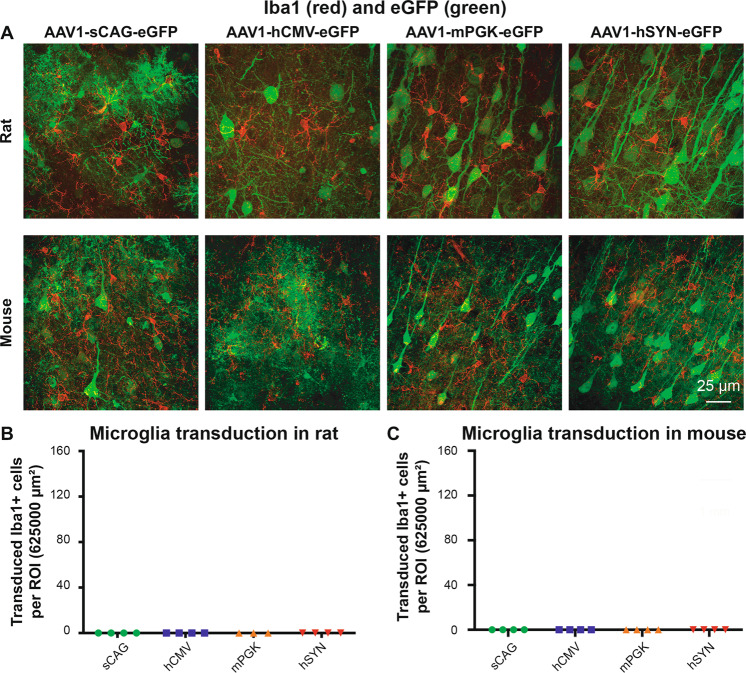
AAV1 with the selected four promoters did not have tropism for microglia. **a** Iba1 (red) and eGFP (green) staining in the cortex of AAV1-sCAG-eGFP, AAV1-hCMV-eGFP, AAV1-mPGK-eGFP and AAV1-hSYN-eGFP injected rats and mice. **b**, **c** Quantification of the number of transduced Iba+ cells in rats and mice. Each dot represents the mean value of one animal. Microscope settings for the eGFP intensity are not identical between the eight experimental groups; the intensity setting for Iba1 is identical throughout the representative images.

The number of transduced microglia was determined by counting the number of Iba1-positive cells that expressed eGFP in the cerebral cortex. The AAV1 serotype together with the promoters sCAG, hCMV, mPGK and hSYN did not result in expression of eGFP in microglia in rats and mice (Fig. 10).

## Discussion

The aim of this study was to characterize expression driven by popular promoters to optimize transduction of layer V corticospinal neurons of the rat and mouse. The viral vector serotype and the promoter are two variables that are known to have a significant effect on the transduction efficiency of AAV vectors. AAV1 is known to be a strong serotype to transduce corticospinal neurons. Subsequently, we examined the impact of the promoter by comparing AAV1 vectors harbouring sCAG, hCMV, mPGK or hSYN promoter. The results show that (1) the mPGK and hSYN promoters activated transgenes in significantly more neurons than the sCAG and hCMV promoters, (2) mPGK and hSYN directed higher levels of transgene expression in neurons than the sCAG and hCMV promoters and (3) the hSYN promoter mediated neuron-specific transgene expression, whereas the sCAG, hCMV and mPGK promoters directed transgene expression in both neurons and non-neuronal cells. These results will be of use for the design of AAV-based gene transfer experiments in corticospinal neurons, which form a widely used fibre tract for studies on long-distance axon regeneration in the injured spinal cord. Table 1 summarizes the cellular transduction profile of AAV1 with the four promoters investigated in this study.

**Table 1:** Cellular tropism of AAV1 with four different promoters.

Cell type	AAV1-sCAG	AAV1-hCMV	AAV1-mPGK	AAV1-hSYN
Cortical neurons	+	+	+++	+++
Layer V corticospinal neurons	+	+	+++	+++
PNN-bearing interneurons	+	+	+++	+++
Oligodendrocyte lineage	++	++	+	−
Astrocytes	++	++	−	−
Microglia	−	−	−	−

Shaded squares indicate that the AAV1 serotype and promoter initiate transgene expression in the mentioned cell type; the plus signs (+) represent the amount of transduced cells for each viral vector. White squares with the minus sign (−) illustrate that no transgene expression was observed in the mentioned cell type.

### Comparison of promoters in cultured cortical neurons after transfection

The functionality of the vector plasmids was validated in cultured cortical neurons by magnetofection prior to AAV production. We choose this transfection method (rather than viral transduction), because this approach is commonly used in primary cortical culture experiments aiming to study individual neurons [29, 30]. The time point of transfection (10 DIV) and analysis (14 DIV) are typically chosen to study matured cortical neurons derived from embryonic day 18 embryos [29, 30]. Interestingly, we did not find a difference in fluorescence intensity between the four promoters in vitro. The short cytosolic protein eGFP is rapidly synthesized after plasmid transfection, which could explain no detectable differences between the promoters for this fluorescent marker at 4 days after transfection. The eGFP intensities in the soma are close to saturation levels, however, we had to use these microscope settings to visualise neurites of the cultured neurons. It is important to note that plasmid transfection is not a delivery method that is comparable with the AAV transduction in vivo; we performed these experiments solely to test plasmid functionality before virus production. The in vitro results are not meant to be predictors for the efficacy in vivo. Furthermore, the cultured cortical neurons are derived from embryonic tissue, which have a high rate of metabolic and transcriptional activity that could allow the ubiquitous activation of various promoters, while this is more selective in adult cortical neurons.

### Analysis pipeline for semi-automated analysis of eGFP+ NeuN+ cells in AAV transduced cortex

Manual analysis of microscope images containing thousands of neurons is time-consuming and prone to human bias and inconsistency. Manually quantifying the number of AAV transduced neurons along with the eGFP fluorescence intensity could therefore be a challenging task. We developed a semi-automated quantification method in the form of a custom analysis pipeline consisting of an ImageJ macro and a data-processing spreadsheet.

The analysis pipeline was compared with manual counts on ten cropped images and we found no statistically significant difference between the two groups (Supplementary Fig. 1), validating the analysis pipeline. However, the ImageJ macro does have some limitations. The user has to set an arbitrary eGFP-to-background intensity ratio that will be used to determine whether a round (NeuN+) cell is eGFP positive or not. This arbitrary threshold is designed to prevent false positives from non-transduced cells that overlap with eGFP-positive branches (e.g. transduced dendrites or astrocytes). We cannot exclude that transduced non-neuronal cells, especially under the AAV1-sCAG and AAV1-hCMV experimental conditions, have resulted in an overestimation in the number of transduced neurons using the analysis pipeline. The ImageJ macro is also limited to the detection of round structures only (e.g. NeuN-positive cells or DAPI and Hoechst stains), and in its current form cannot be applied to detect more complex morphologies such as astrocytes, microglia and PNNs.

### The AAV1 serotype for transduction of the corticospinal tract and cortex

The AAV1 serotype is commonly used to transduce the CST via intracortical injections [31–38]. An earlier study that compared seven AAV serotypes (AAV1–AAV6, and AAV8) identified AAV1 and AAV5 as the best and second-best serotypes to transduce the CST in rats [20]. Consistent with that study, we found in both mice and rats that AAV1 transduces almost twice as many cortical neurons and layer V corticospinal neurons as AAV5 (Supplementary Figs. 2, 3), highlighting that AAV1 is superior to AAV5 for transduction of the CST and cortex. We also observed that AAV1 harbouring an hSYN promoter directs a stronger expression of eGFP than AAV5 with this promoter (Supplementary Figs. 2, 3). This difference may be caused by the entry of more copies of the AAV1 than the AAV5 vector into cortical neurons. Both AAV serotypes bind to α2–3 and α2–6 N-linked sialic acids [39–42], but their endocytosis requires different cellular co-receptors. For instance, AAV1 interacts with integrins [43], while AAV5 uses platelet-derived growth factor receptor as co-receptor for transduction [24]. Furthermore, AAV1 and AAV5 bind to different domains of the adeno-associated virus receptor on the cell surface to initiate transduction [44]. The endosomal processing of the viral vector particles inside the cell, and de-capsidation and release of the viral genome into the cell nucleus is also different between the two serotypes [45–47].

An alternative approach to transduce the CST is by using AAVs that have retrograde transduction properties and inject them directly into the spinal cord. AAV6 [48] and a directed evolved variant of AAV2 named ‘AAV2-Retro’ [49] have the capability for retrograde transduction in the CNS. AAV2-retro has been applied for robust transduction of the CST by injection into the spinal cord of adult mice [50, 51].

### Comparison of four promoters for transgene expression

This study consists of a direct side-by-side comparison between AAV1 vectors harbouring four compact promoters for their ability to drive transgene expression in the CST and cortex. The in vivo findings for each promoter are discussed below.

The hSYN promoter resulted in neuron-specific transgene expression and this corroborated previous studies using this promoter [21, 33, 52–57]. One study reported that the synapsin promoter is the most selective promoter among five neuron-specific promoters that were tested in the rat brain [56]. We found that the hSYN-driven transgenes were activated in various subpopulations of cortical neurons, as we observed expression in multiple cortical layers including layer V pyramidal neurons and PNN-expressing neurons. The hSYN promoter is also one of the experimental groups with the strongest eGFP expression in vivo. To our knowledge, there is no promoter available that is specific for layer V pyramidal neurons that give rise to the CST. Thus, the hSYN promoter is currently the promoter of choice to drive transgene expression in corticospinal neurons, because it is small (allowing large coding sequences to be inserted), strong and neuron specific.

We found that the mPGK promoter in AAV1 directed strong neuronal expression and limited transgene expression in oligodendrocytes. Others have found relatively similar results; a lentivirus with the mPGK promoter injected into the sensorimotor cortex [58], hippocampus [59], striatum [59] and substantia nigra [60, 61] confirmed that this promoter preferentially expresses in neurons rather than non-neuronal cells. In contrast to our study using AAV1, one of the lentivirus studies reported low tropisms for astrocytes using the mPGK promoter [61]. A study that used the human PGK promoter with AAV serotypes 6, 9, and rh10 reported predominant expression in neurons and a limited number of astrocytes and oligodendrocytes [62, 63]. In addition, we found that the mPGK promoter is a strong promoter in terms of transgene expression in the sensorimotor cortex; mPGK promoter was superior over the hCMV and sCAG promoters. Consistently, a study that compared AAV2 vectors containing the PGK and CMV promoters in the mouse retina also observed that PGK was the stronger promoter between the two [64].

The sCAG and hCMV promoters in AAV1 had relatively weak transgene expression. These promoters could therefore be preferable for studies that require moderate to low transgene expression in the CNS. The relatively low expression by CMV and CAG promoters in some brain regions has been reported before [65–67]. More specifically, the CMV promoter has been reported to be silenced over time with AAVs in piriform cortex [65], hippocampus [65, 66] and substantia nigra [67]. It has also been reported for the chicken β actin promoter, a large part of this DNA sequence is identical to the sCAG promoter, to express poorly in motor neurons of the spinal cord [68]. The relatively poor expression by the hCMV promoter in cortical neurons could be caused by transcriptional inactivation caused by epigenetic factors [69–71]. Investigation of expression levels at an earlier time point and potential epigenetic silencing could shed some light on this. However, such investigations were beyond the scope of this study. Importantly, the promoters sCAG and hCMV directed the expression of transgenes in astrocytes, oligodendrocytes and cortical neurons including layer V corticospinal neurons. The ubiquitous sCAG and hCMV may therefore not be the ideal promoters for studies aiming to transduce cortical neurons only. Many studies support our findings that the hCMV and sCAG promoters transduce astrocytes, oligodendrocytes and a restricted number of cortical neurons in vivo. For instance, the transduction of these cell types by the AAV1 serotype with the CMV and CAG promoters have also been observed by others [20, 21, 72]. Lentivirus containing the hCMV and full-length CAG promoter also transduced neurons, astrocytes and oligodendrocytes when injected into the striatum [73]. The CMV promoter has been reported to transduce astrocytes with high efficiency when combined with AAV6 and AAV8 [23, 74]. The CMV promoter has also been reported to activate transgenes in astrocytes, oligodendrocytes and neurons with AAV5 [57], AAV8 [20] and AAV9 [75]. Another study reported that the CAG promoter transduces these cell types with the AAV8 serotype as well [76].

Microglia have been proven to be challenging to transduce with AAVs in vivo (reviewed in [77]). We found that the AAV1 serotype together with the promoters sCAG, hCMV, mPGK and hSYN did not result in transgene expression in microglia. AAV2 harbouring a CMV promoter has been reported before to be unable to activate transgenes in microglia [78]. Yet, AAV-mediated transduction of microglia has been reported with a promoter derived from the macrophage marker F4/80 or CD68 gene [79–81]. This indicates that transduction of microglia is rare using the common promoters with AAVs, and successful transgene activation in these cells may require the identification of microglia-specific promoters or another type of viral vector for transgene delivery.

The current experiments do not shed light on the reason why the mPGK and hSYN promoters drive high levels of transgene expression and why the sCAG and hCMV promoters are significantly weaker. The mPGK and hSYN promoters contain regulatory elements that are recognized by a combination of cellular transcription factors that are normally involved in regulating the expression of the phosphoglycerate kinase and synapsin genes in neurons. One might therefore speculate that cellular promoters have an advantage over viral-derived promoters because they act in their natural context whereas viral promoters are activated by (a set of) transcription factors that may not be optimal drivers of transcription.

## Conclusions

It is important for any gene therapy that transgenes are delivered with high cell specificity and at adequate expression levels. This study investigated the activity of four promoters in a side-by-side comparison in mice and rats at 6 weeks after AAV injection. The AAV1 serotype exhibited neuronal tropism with all four investigated promoters. Yet, the choice of promoter influences: (1) the strength of transgene expression; and (2) the type of cells that will express the transgene of interest (Table 1).

AAV-mediated gene transfer for spinal cord injury research will require robust expression of regeneration-associated genes in neurons of the CST. The mPGK promoter and hSYN promoters resulted in strong transgene expression, while the commonly used hCMV and sCAG had relatively weak expression in cortical neurons.

High cell specificity for layer V corticospinal neurons is crucial to avoid unwanted side effects when delivering regeneration-associated genes. These could be caused, for instance, if the transgene alters gene expression in glia cells. It is unclear how AAV-mediated gene transfer of regeneration-associated genes will influence neurons other than layer V corticospinal neurons. Manipulation of PNN-bearing interneurons potentially affects the regeneration response of layer V corticospinal neurons after injury. For example, it has been shown that activation of interneurons in the retina (amacrine cells) restrict neuronal survival and axon regeneration of retinal ganglion cells after optic nerve injury [82].

A promoter with robust and exclusive expression in layer V corticospinal neurons is desired for spinal cord injury experiments; however, to date, such a promoter has not yet been identified. In this study, we showed that the hSYN promoter has strong transgene expression and is specific to neurons with no expression in glia cells. Based on the data presented here, the AAV1 viral vector serotype and the hSYN promoter is the optimal combination for transgene delivery to the CST of mice and rats.

## Supplementary information

Supplementary Table 1

Supplementary Figure 1

Supplementary Figure 2

Supplementary Figure 3

Supplementary Figure 4

Supplementary information

Related Manuscript File
