# Supplementary material for: Optimization of adeno-associated viral vector-mediated transduction of the corticospinal tract: comparison of four promoters
Source: Gene Ther. 2020 Jun 23;28(1):56–74. doi: 10.1038/s41434-020-0169-1 (PMC7902269; doi:10.1038/s41434-020-0169-1)
Supplement: Supplementary file 1 — Supplementary Table 1 [file 41434_2020_169_MOESM1_ESM.docx]

| Line | ImageJ Macro |
| --- | --- |
| 1  2  3  4  5  6  7  8  9  10  11  12  13  14  15  16  17  18  19  20  21  22  23  24  25  26  27  28  29  30  31 | //preamble - modify image as appropriate  //select parameters ‘area’ and ‘median’ in set measurements as ImageJ output  run("Set Scale...", "distance=0 known=0 pixel=1 unit=pixel");  waitForUser("draw a RECTANGULAR area of interest and hit ok");  run("Clear Outside");  run("Duplicate...", " ");  setRGBWeights(1, 0, 0);  run("8-bit");  //code representing the red channel  setAutoThreshold("Otsu dark");  setOption("BlackBackground", false);  run("Convert to Mask");  run("Watershed")  run("Size...") //Change the size of your image here to match the open file  run("Make Binary");  run("Analyze Particles...", "size=100-3500 circularity=0-1.00 show=Outlines exclude summarize add");  close;  close;  setRGBWeights(0, 1, 0);  run("8-bit");  run("Size...");  roiManager("Show all");  roiManager("Select All");  waitForUser("happy with the selection?");  roiManager("Measure");  //code representing the output of the green channel  //make sure that the measurement tab is selected  saveAs("Results");  roiManager("Delete");  run("Clear Results");  close; |
