# Supplementary figures and images for: Optimization of adeno-associated viral vector-mediated transduction of the corticospinal tract: comparison of four promoters
Source: Gene Ther. 2020 Jun 23;28(1):56–74. doi: 10.1038/s41434-020-0169-1 (PMC7902269; doi:10.1038/s41434-020-0169-1)

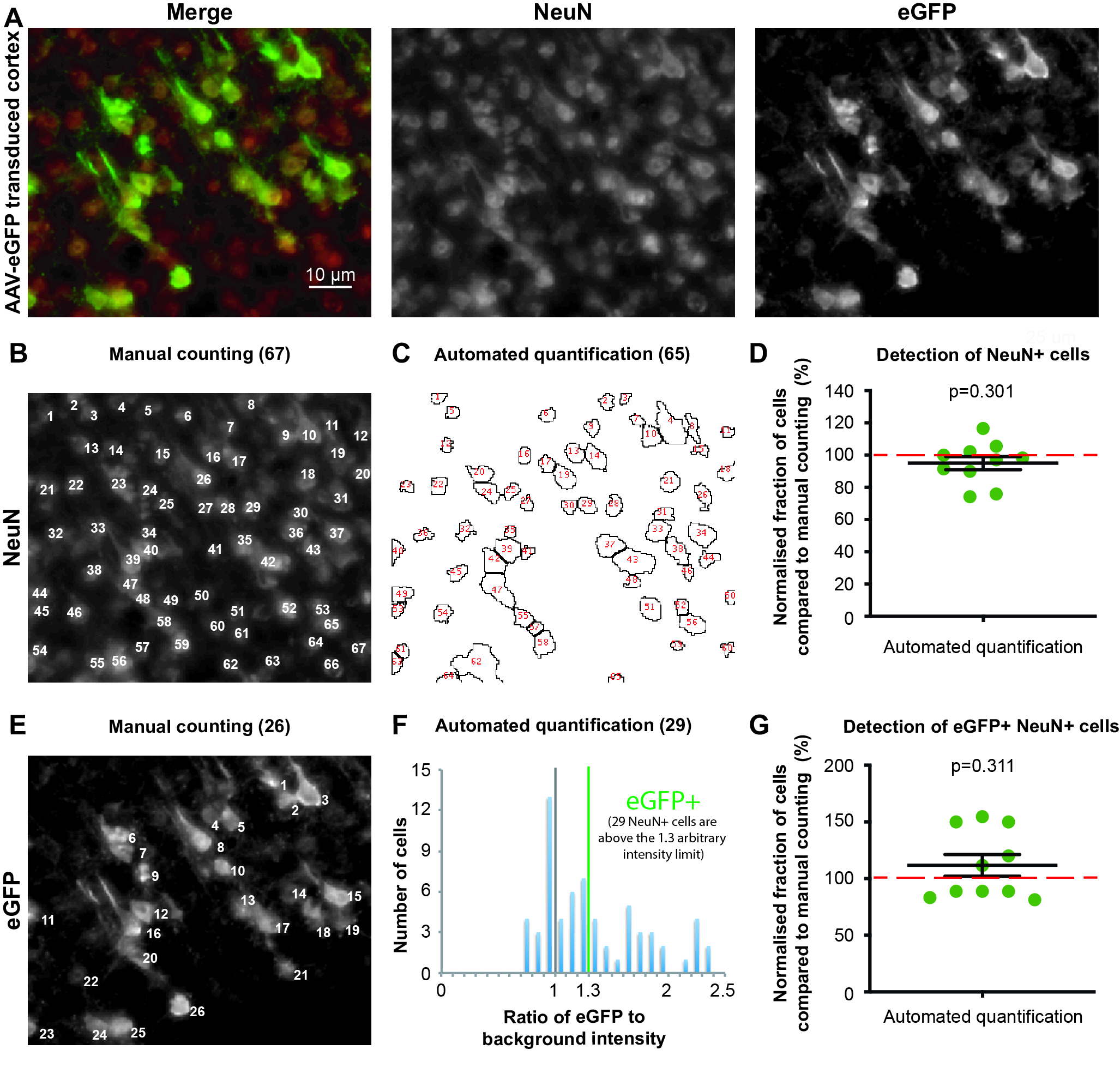

Supplement: Supplementary file 2 — Supplementary Figure 1 [file 41434_2020_169_MOESM2_ESM.tif]

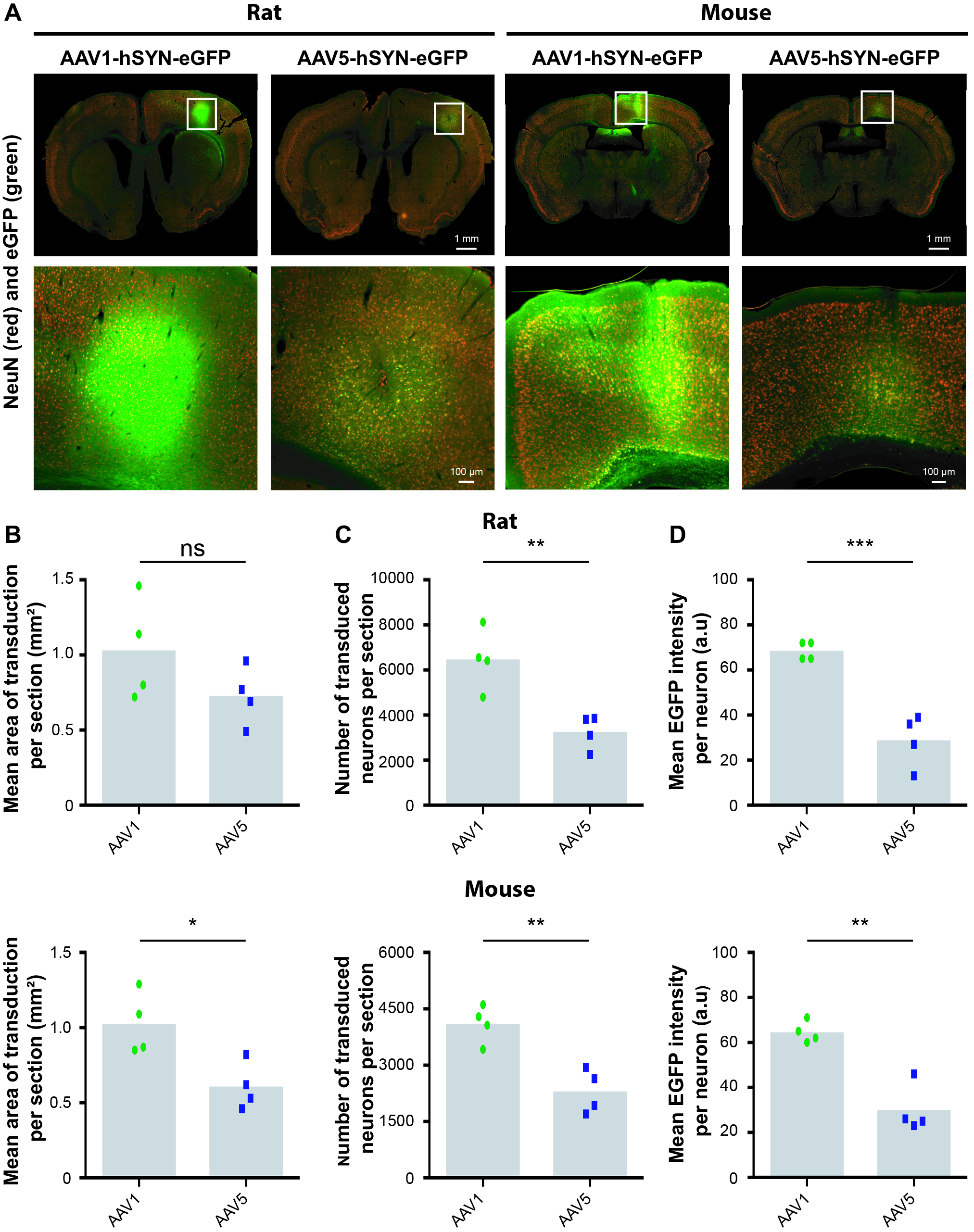

Supplement: Supplementary file 3 — Supplementary Figure 2 [file 41434_2020_169_MOESM3_ESM.tif]

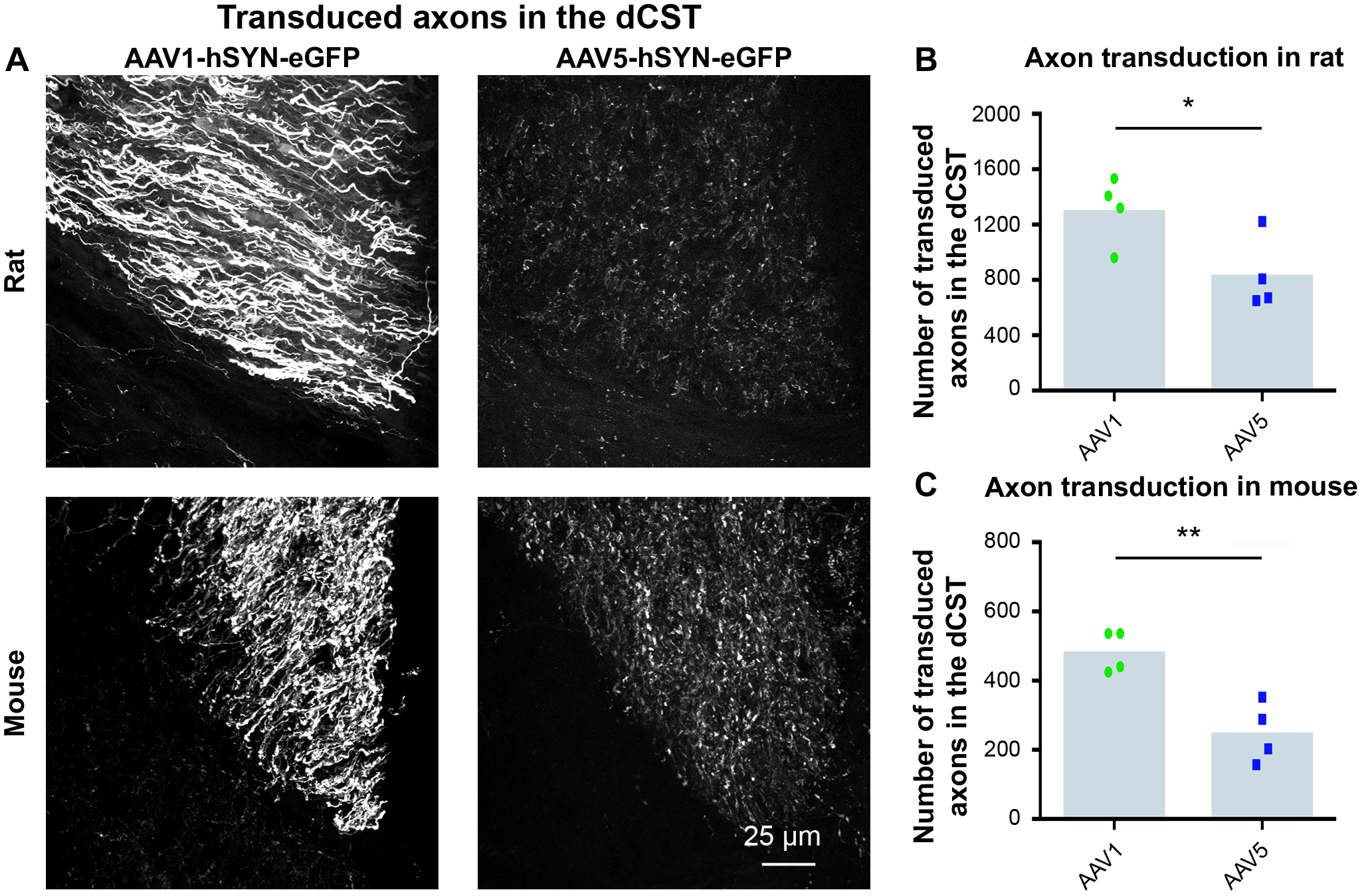

Supplement: Supplementary file 4 — Supplementary Figure 3 [file 41434_2020_169_MOESM4_ESM.tif]

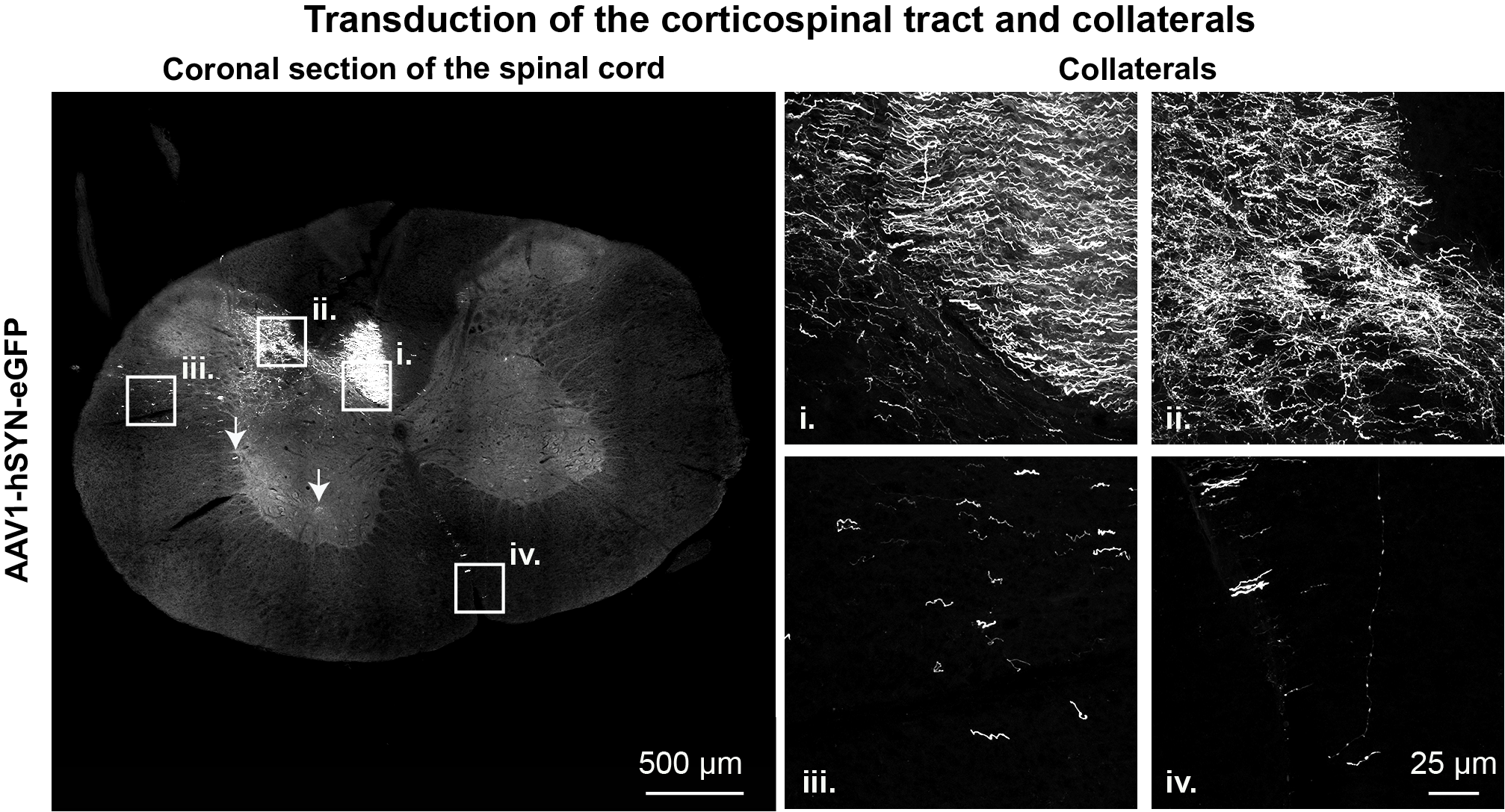

Supplement: Supplementary file 5 — Supplementary Figure 4 [file 41434_2020_169_MOESM5_ESM.tif]
