# Supplementary material for: Optimization of adeno-associated viral vector-mediated transduction of the corticospinal tract: comparison of four promoters
Source: Gene Ther. 2020 Jun 23;28(1):56–74. doi: 10.1038/s41434-020-0169-1 (PMC7902269; doi:10.1038/s41434-020-0169-1)
Supplement: Supplementary file 6 — Supplementary information [file 41434_2020_169_MOESM6_ESM.docx]

**Supplementary Table 1** ImageJ macro for detection of cells in the red channel and measuring the fluorescent intensity in the green channel.

The code can be copied into an ImageJ Marco for usage. The lines starting with ‘//’ are comments for the user. **Lines 1-3**: the macro begins by removing the scale and therefore all input and output will be in pixels. **Lines 4-6**: Create a region of interest (roi) within the open image. A duplicate is made so that the macro can process any images regardless of the image name. **Lines 7-9**: The macro will open the red channel (e.g. immunofluorescence for cell marker NeuN). **Lines 10-15**: The macro will create a mask on the cells positive for the marker in the red channel using the standard ImageJ thresholding called ‘Otsu’. Note that the thresholding parameters were manually determined by the user for this experiment, and should be re-determined for each new experiment. Two cells that are close to each other will be separated using the tool watersheding. It is important to make a note of the image size of the newly drawn region of interest, which was created in lines 4-6, in the pop-up screen (The image size in pixel is written in the top left corner). **Lines 16-18**: The particle analysis is important because it identifies cells based on their size. The current settings will exclude particles from the red channel that are smaller than 100^2^ pixels and bigger than 3500^2^ pixels. After cell identification, the masks are stored in the roi manager and the red channel gets closed. **Lines 19-27**: The masks, which contain cells that are positive for the marker in the red channel, are moved to the green channel containing eGFP positive cells. The user can view the overlay on the green cells and when satisfied press ‘okay’ to measure the eGFP intensities. **Lines 28-31**: The output of the ImageJ Macro is the ‘number of red cells’, the ‘area surface of each red cell’ and the ‘median eGFP intensity of each red cell’. The output can be stored in a data-processing spreadsheet and the Macro will be closed. An automated data-processing spreadsheet (see **Supplementary Data 1**) can next be used to determine the number of eGFP+ red channel marker+ cells based on a background intensity measurement of the green channel.

**Supplementary Data 1** Data-processing spreadsheet for ImageJ macro output

**Supplementary Figure 1** Detection of NeuN+ and eGFP+ NeuN+ cells in AAV-transduced mouse cortex via manual analysis and a new custom analysis pipeline. Adult mice were stereotactically injected with AAVs expressing eGFP and fixed by perfusion after six weeks. Immunohistochemistry was performed to visualize NeuN (red) and eGFP (green) in the cortex. A cropped image of an AAV-transduced cortical area is shown in (A). The number of NeuN+ cells was manually counted in (B) and detected by the ImageJ macro in (C). (D) Quantification of the number of NeuN+ cells by manual counting and the ImageJ macro (P>0.05, N=10 per group, one-sample Wilcoxon signed rank test for median = 100). (E) Manual analysis of the number of eGFP+ NeuN+ cells. (F) Quantification of the number of eGFP+ NeuN+ cells using the analysis pipeline. The eGFP intensity of all previously detected NeuN+ cells were measured and divided by the intensity of the background within the region of interest to obtain a eGFP/background intensity ratio. The histogram shows the eGFP/background intensity ratio on the X-axis and the frequency (number of cells) on the Y-axis. The grey vertical line represents an eGFP/background ratio of 1 and therefore the number of NeuN+ cells with eGFP intensity similar to the average background intensity (e.g eGFP- NeuN+ cells). Since measuring fluorescent intensities will follow a normal distribution, it is important to set an arbitrary limit for measuring eGFP+ NeuN+ cells. The green vertical line represents an eGFP/background ratio of 1.3 (e.g. 30% above the average background intensity) and was determined to be a good threshold to detect eGFP+ NeuN+ cells. (G) Quantification of the number of eGFP+ NeuN+ cells by manual counting and the ImageJ macro (P>0.05, N=10 per group, one-sample Wilcoxon signed rank test for median = 100). Data is shown as average ± SEM. The red dashed line in panels D and G represent the expected automated quantification performance (100% relative to manual counts). Images were taken with a Zeiss AxioScan Z1 microscope with 20x objective.

**Supplementary Figure 2** Transduction of AAV1 and AAV5 harbouring the hSYN-eGFP expression cassette in the sensory-motor cortex. (A) NeuN+ and eGFP staining in the cortex of AAV1-hSYN-eGFP and AAV5-hSYN-eGFP injected rats and mice. (B) Quantification of the mean area of transduction in 40 μm thick sections (Rat, df=6, t=1.55, P>0.05, Student’s t-test; Mouse, df=6, t=3.22, P<0.05, Student’s t-test). (C) Quantification of the number of transduced NeuN+ cells. eGFP+ NeuN+ cells were detected using the analysis pipeline and neurons were considered as transduced when the eGFP intensity was 1.3x higher than the eGFP to background intensity ratio (Rat, df=6, t=4.13, P<0.01, Student’s t-test; Mouse, df=6, t=4.66, P<0.01, Student’s t-test). (D) Quantification of the mean eGFP intensity per transduced neurons (Rat, df=6, t=6.44, P<0.001, Student’s t-test Mouse, df=6, t=5.87, P<0.01, Student’s t-test). The grey bars depict the averages and each dot represents the mean value of one animal. A total of 4 animals were analysed for each condition. Images were taken with identical microscope settings between the experimental groups. Ns, not statically significant; * P<0.05; ** P<0.01; *** P<0.001.

**Supplementary Figure 3** Transduction efficiency of AAV1 and AAV5 in layer V cortical neurons measured by quantifying their axons in the spinal cord. (A) Representative images of transduced axons in the left dorsal column of the spinal cord at level C4 of AAV1-hSYN-eGFP and AAV5-hSYN-eGFP injected rats and mice. The axons in the AAV1 group were brighter than the AAV5 group. (B - C) Quantification of the number of transduced axons in rats (df=6, t=2.58, P<0.05, Student’s t-test) and mice (df=6, t=4.43, P<0.01, Student’s t-test). The grey bars depict the averages and each dot represents the axon count of one animal. A total of 4 animals were analysed for each condition. Images were taken with identical microscope settings between the experimental groups. * P<0.05; ** P<0.01.

**Supplementary Figure 4** Transduction of the CST in a rat injected with AAV1-hSYN-eGFP. The section at the cervical level of the spinal cord was stained for eGFP. eGFP-positive axons are descending in the dorsal column and collaterals are projecting towards the lamina of the spinal cord; insets i-iii and arrows. Transduced axons in the ipsilateral ventral CST are visible close to the midline of the cervical spinal cord, inset iv. The overview image was taken with a tile-scanning epifluorescence microscope, while the zoom-in pictures were taken using a confocal microscope.
